# xMAN: extreme MApping of OligoNucleotides

**DOI:** 10.1186/1471-2164-9-S1-S20

**Published:** 2008-03-20

**Authors:** Wei Li, Jason S Carroll, Myles Brown, X Shirley Liu

**Affiliations:** 1 Department of Biostatistics and Computational Biology, Dana-Farber Cancer Institute; Department of Biostatistics, Harvard School of Public Health, Boston, MA, USA; 2 Department of Medical Oncology, Dana-Farber Cancer Institute, Harvard Medical School, 44 Binney St, Boston, MA 02115, USA

## Abstract

**Background:**

The ability to rapidly map millions of oligonucleotide fragments to a reference genome is crucial to many high throughput genomic technologies.

**Results:**

We propose an intuitive and efficient algorithm, titled extreme MApping of OligoNucleotide (xMAN), to rapidly map millions of oligonucleotide fragments to a genome of any length. By converting oligonucleotides to integers hashed in RAM, xMAN can scan through genomes using bit shifting operation and achieve at least one order of magnitude speed increase over existing tools. xMAN can map the 42 million 25-mer probes on the Affymetrix whole human genome tiling arrays to the entire genome in less than 6 CPU hours.

**Conclusions:**

In addition to the speed advantage, we found the probe mapping of xMAN to substantially improve the final analysis results in both a spike-in experiment on ENCODE tiling arrays and an estrogen receptor ChIP-chip experiment on whole human genome tiling arrays. Those improvements were confirmed by direct ChIP and real-time PCR assay. xMAN can be further extended for application to other high-throughput genomic technologies for oligonucleotide mapping.

## Background

The ability to rapidly map millions of oligonucleotide fragments to a reference genome is crucial to many high throughput genomic technologies. For example, Affymetrix, Nimblegen, and Agilent have recently developed oligonucleotide arrays to tile all the non-repetitive genomic sequences of complex eukaryotic genomes. Since the arrays were usually designed based on an older genome assembly, it is important to remap all the probes to the newest genome assembly or transcriptome annotation during data analysis [1], under the assumption that the current genome and transcriptome are more precise than the earlier ones. It is not uncommon for a probe to map to multiple locations in the genome. As a result, the probe could give rise to unexpected behaviour if information on its genome copy number is unknown or ignored. Other examples are the next-generation sequencing approaches to annotate novel transcripts [2,3] and regulatory elements [4]. In these applications, millions of short oligonucleotide fragments are generated and mapped to a reference genome to identify the transcript or *cis*-element clusters.

The current algorithms available for fast sequence similarity search such as BLAST [5], MegaBLAST [6], BLAT [7] and MUMmer [8] were not specifically designed for mapping millions of query sequences. As a result, using such algorithms often take very long time, even on computer clusters with fast processors and big memory. For example, in the recent MicroArray Quality Control project [9], it took approximately two days to map the half a million probes on the Affymetrix U133 expression microarray to the RefSeq mRNA database of approximately 70 MB size on a Beowulf cluster with 248 AMD Opteron Dual Processor nodes. This translates into inhibitive time and resources required to map the 42 million probes on Affymetrix genome tiling microarrays to complete mammalian genomes.

We propose an intuitive and efficient method xMAN (namely extreme MApping of OligoNucleotide) for the rapid mapping of millions of query oligonucleotide fragments to the reference genome of any given length. xMAN differs significantly from existing algorithms. First, instead of indexing the reference genome which is memory expensive, xMAN transforms all the query sequences into integers and stores them in RAM as a hash table. Secondly, when scanning through a genome of any size for query mapping, xMAN hinges on bit shifting operation over sliding windows to boost its search speed. In this paper, we will explain xMAN's underlying algorithm, compare its performance with other methods, and discuss its application in tiling microarray data analysis.

## Results

### Tiling microarray probes remapping

Tiling microarrays have probes that cover essentially the entire non-redundant genome in an unbiased fashion. Such arrays have diverse applications, including chromatin-immunoprecipitation coupled with DNA microarray analysis (ChIP-chip), comparative genome hybridization, empirical detection of novel transcripts and polymorphism discovery [10]. The average nucleotides spacing between centres of neighbouring probes defines the tiling ‘resolution’. There are several tiling array platforms with different probe length, resolution, and manufacturing characteristics. We focus our study on Affymetrix tiling arrays since they have the highest probe density, with approximate 42 million 25-mer probes covering the non-repetitive human genome at 35 bp resolution. The sheer amount of raw data generated on these arrays poses challenges for data analysis.

Despite extensive efforts to design statistical algorithms to analyze Affymetrix tiling microarray [11-16], potential probe mapping problems exist and might have serious downstream consequences. Early assessments [1] on Affymetrix expression arrays revealed that remapping probe sets to the newest genome could create as much as 50% discrepancy in predicted differentially expressed genes, regardless of the analysis methods used. Therefore, it is crucial to remap all the tiling array probes to the current genome to ensure more precise downstream analyses. Furthermore, one primary objective in microarray analysis is to minimize probe cross-hybridization. Most tiling microarrays are designed based on repeat-masked genome, which still contains many repetitive elements including tandem repeats [17] with period longer than 12 bp and segmental duplications [18]. Affymetrix tiling probe selection operates in a local fashion, which does not check whether a probe matches elsewhere in the whole genome (S. Cawley, *pers. commun*.). It does map all the probes to the genome afterwards, although only to the repeat-masked genome which could be problematic. This approach also sometimes maps the same probe to multiple locations in a short genomic region. When calculation on the region is performed assuming all the probe measures are independent, this mapping is likely to inflate the p-value of the region and create a false positive. These problems could be potentially addressed by taking into account each probe's copy number [14] and filtering out repetitive probes [15] in the analysis.

**Figure 1:**
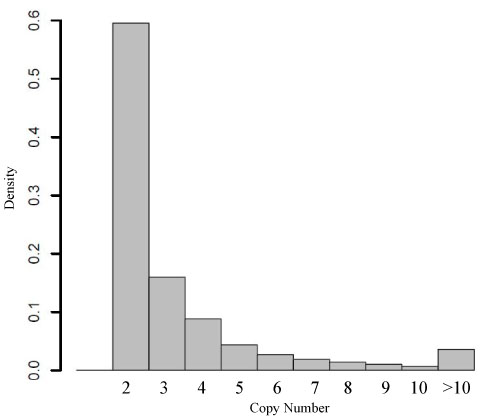
**Copy number histogram of ~42 million probes on the Affymetrix human genome 1.0 tiling arrays.** Only probes with more than one match in the genome are shown.

Affymetrix Human Tiling 1.0 arrays were designed based on build NCBIv34 of the human genome, and we downloaded the Affymetrix probe mapping (BPMAP) files from . We used xMAN to remap the ~42 million probes to build NCBIv35 of the human genome, in both Watson and Creek strands without repeat-masking (designated as xMAN BPMAP). xMAN stores the number of times a probe's 25-mer sequence maps to the genome, so as to aid probe cross-hybridization estimation. The whole process took less than six hours on an AMD Opteron single-CPU Linux computer, including importing probe sequences into hash table, scanning the genome, and writing the result BPMAP file. There are a total of 41,370,900 unique 25-mers on the whole human tiling microarrays, among which 301,947 have been synthesized on the arrays multiple times, resulting in a total of 41,782,720 array spots (Table 1). An unexpected observation is that 13,120 (0.03%) probes no longer maps to the NCBIv35 genome, thereby should be excluded from downstream analysis. Another surprise is that although the tiling probes are selected from the ‘repeat-masked’ genome, 1,215,226 (2.94%) of the probes have multiple genomic copies (Fig. 1). For example, one probe with sequence TCGGCCTCCCAAAGTGCTGGGATTA, which was designed to interrogate a non-repetitive sequence on chromosome 19, mapped to 114,450 locations in the genome. It is worth noting that a typical transcription factor only binds less than 1% of the genome. Thus, the ~3% probes with multiple genome copies could bring substantial influence to a ChIP-chip analysis.

**Table 1:** xMAN probe mapping of various Affymetrix tiling arrays to the most recent genome assembly

Affymetrix tiling arrays	#UniqSeq	#Seq. MEntries	#Query Entries	#Seq. MGenomeMatches	#Seq.NoGenomeMatch	#Total Entries
Human ENCODE 1.0^a^	721,043	14,322	756,555	16,444	506	884,634
Human Chr21/22^a^	979,553	21,930	1,054,324	49,316	75	1,627,746
Human Promoter^b^	4,220,999	40,099	4,275,079	251,460	2,537	5,706,819
Human Tiling 1.0 & 2.0^b^	41,370,900	301,947	41,782,720	1,215,226	13,120	48,332,137
Mouse Tiling 1.0 & 2.0^c^	38,788,060	431,551	39,576,383	993,890	437,877	51,036,801
Mouse Promoter^c^	4,096,798	30,835	4,154,546	192,119	37,483	5,716,068
Arabidopsis thaliana 1.0^d^	3,046,178	7,275	3,053,686	164,728	0	3,772,912

Table head line:

#UniqSeq: Number of unique 25-mer in the original BPMAP file

#Seq.MEntries: Number of 25-mer with multiple spots in the array

#QueryEntries: Number of array spots in the original BPMAP file

#Seq.MGenomeMatches: Number of 25-mer with multiple genomic copies

#Seq.NoGenomeMatch: Number of 25-mer with no match in the genome

#TotalEntries: Number of total entries in the xMAN mapping

^a^The original Affymetrix probe mapping is from the NCBIv33 human genome. The new xMAN probe mapping is based on NCBIv35 human genome.

^b^The original Affymetrix probe mapping is from the NCBIv34 human genome. The new xMAN probe mapping is based on the NCBIv35 human genome.

^c^The original Affymetrix probe mapping is from the NCBIv33 mouse genome. The new xMAN probe mapping is based on the NCBIv35 mouse genome.

^d^Both the original Affymetrix probe mapping and xMAN probe mapping are based on the TIGRv5 Arabidopsis genome.

Similar situation was observed when xMAN was applied to remap several other Affymetrix tiling arrays for human, mouse and Arabidopsis (Table 1).

Based on the above observation, xMAN used the following rules to remove probe redundancy in the human genome tiling array BPMAP: 1) all the probes with copy number more than 10 are filtered out; 2) the same 25-mer is mapped only once within a 1 kb window along the genome; 3) the remaining probes with multiple copy numbers are not mapped unless it is at least 30bp apart from the previous probe (the average probe spacing for the array is 35 bp). The correct whole genome probe copy number is stored with each probe's 25-mer sequence, regardless of whether it is mapped to a certain region or not by rules 2) and 3).

### Comparison with existing tools

Many tools are available for sequence similarity search, among which BLAST [5], MegaBLAST [6], BLAT [7] and MUMmer [8] are probably the most widely used. We compared the performance of xMAN with these tools in the same Linux computer to map the 42 million human genome tiling probes to the human genome. BLASTN [5] scans for short matches (usually 11-mer) in the genome and extends those matches into high-scoring pairs (HSPs). MegaBLAST utilizes a greedy algorithm [6] to search for nucleotide sequence alignment and is optimized for aligning slightly different sequences. BLAT [7] indexes non-overlapping K-mers in the genome and hashes them inside the computer RAM, then scans linearly through the query sequence. MUMmer [8] adopts a suffix tree data structure for rapid sequence alignment, but is memory intensive. MUMmer 3.0 uses approximately 17 bytes for each nucleotide in the reference genome, thus requires ~51 GB (3 G* 17) of RAM to create the human genome data structure. We do not have access to a computer that meets this RAM requirement, thus did not include MUMmer in our comparison.

It took BLAST 2,482 minutes, BLAT 19 minutes, and MegaBLAST (with an optimal word size of 20) 0.8 minute to search the first 10 thousand probes against the human genome. Since search time is approximately proportional to the query size, extrapolating these numbers predicts that the three algorithms will take about 173,740, 1,330 and 56 CPU hours to map all 42 million probes, respectively. MegaBLAST appears to be a proper solution to this specific probe-mapping problem, and is extremely efficient with this word size of 20. Nevertheless, its advantage diminishes when mapping shorter fragments with smaller word size. For instance, MegaBLAST requires almost 2 CPU minutes to search 10 thousand 18-mer probes against the genome with word size of 12, i.e. 140 CPU hours for the 42 million 18-mer probes. In comparison, word size in xMAN, which is the minimal length of an identical match, is always equal to the length of the query oligonucleotide and has no effect on the searching time. In any case, xMAN needs less than 6 CPU hours in completing the 42 million probe mapping, and is at least an order of magnitude faster than other popular algorithms.

### Impact of the updated probe mapping on the tiling array analysis

We aim to systemically investigate the impact of the updated probe mapping on tiling array data analysis. We recently developed a Model-based Analysis of Tiling arrays (MAT) algorithm [14] to reliably detect ChIP-enriched regions on Affymetrix tiling arrays. MAT employs a linear model to estimate the baseline probe behaviour based on probe sequence and copy number. To our knowledge, it is the only algorithm that considers probe copy number information in tiling array analysis. As our goal here is to assess the pure effect of probe mapping on tiling array data analysis, we only used MAT to investigate the impact of xMAN probe mapping. Since the Affymetrix BPMAP does not contain probe copy number information, we used 1 copy for every probe.

**Figure 2:**
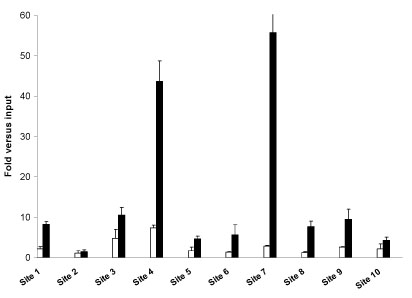
**Characterization of ER binding sites identified only through xMAN probe mapping**. Standard ChIP assays of ER were performed with anti-ER antibody. Immunoprecipitated DNA was quantified by qPCR using primers spanning 10 randomly selected regions identified by MAT only with xMAN probe mapping. The results are shown as vehicle (control, white bars) or estrogen (black bars) fold enrichment over input and are the average of three replicates±SE. The 10 regions are provided as NCBIv35 chromosomal coordinates: Site 1(chr10:94549192-94550690), Site 2(chr11:46253821-46255330), Site 3(chr11:100812061-100813479), Site 4(chr4:188058695-188060091), Site 5(chr5:52253648-52254807), Site 6(chr5:133383322-133384523), Site 7(chr7:150991115-150992368), Site 8(chr8:88996063-88997399), Site 9(chr8:99413611-99415262), Site 10(chr8:102555348-102556385).

We applied MAT to the estrogen receptor (ER) whole genome ChIP-chip data [19] using both Affymetrix and xMAN BPMAPs (Table 2). Regardless of the thresholds, the consistency between the ChIP-regions from the two probe mappings is usually around 95%. Our analysis suggested that at the same false discovery rate (FDR: expected percentage of false positives in a set of predictions) threshold, xMAN BPMAP can significantly increase the number of detected ChIP-regions compared to Affymetrix BPMAP. At 0% FDR threshold, MAT identified 635 (24%) more regions with xMAN BPMAP, among which 123 could not be identified even at 5% FDR using Affymetrix BPMAP. We randomly selected 10 out of the 123 xMAN-specific regions to conduct site-specific ER ChIP and real-time quantitative PCR assay (qPCR), and found 9 out of the 10 regions to be ChIP-enriched (> 4 fold) in an estrogen-dependent manner (Fig. 2). This indicated that most of the xMAN-specific ER binding sites are real, suggesting a reduced false negative rate in the analyses using xMAN BPMAP. On the contrary, only 53 regions were found at 0% FDR using Affymetrix BPMAP but not found using xMAN BPMAP at 5% FDR. All 53 Affymetrix-specific ChIP-regions reside in repetitive sequences, suggesting that they are most likely false positives, due to the inflated signals on the repetitive probes in Affymetrix BPMAP.

**Table 2:** Whole genome ER ChIP-chip results based on either Affymetrix or xMAN probe mapping under different FDR thresholds

FDR thresholds (%)	0	1	2	5
Affymetrix^a^	2,646 (2,312)	5,221 (4,572)	5,714 (4,993)	7,293 (6,413)
xMAN^a^	3,281 (2929)	6,544 (5,820)	7,563 (6,760)	8,890 (7,925)
Shared Regions^b^	2,475 (2,217)	5,006 (4,481)	5,436 (4,876)	6,871 (6,184)
Percentage of Shared Regions^c^	93.5 (95.9)	95.9 (98.0)	95.1 (97.6)	94.2 (96.5)

^a^The numbers of ChIP-regions identified by MAT are shown in the table. A ChIP-region is annotated as repeat if more than 70% of the region is within RepeatMasker repeats, simple repeats, or segmental duplications. The numbers of non-repeat regions are shown in the parentheses.

^b^ChIP-regions identified from Affymetrix_NCBIv34 probe mapping were converted into NCBIv35 version using LiftOver program (). Two regions are considered the same if they overlap by more than 50%.

^c^The percentage of shared regions was defined as the number of shared regions divided by the number of regions identified using Affymetrix probe mapping.

**Figure 3:**
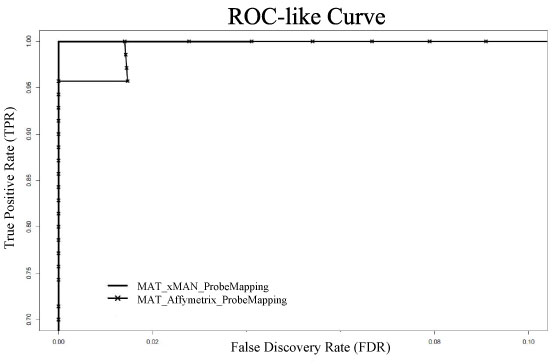
**ROC-like curve for ENCODE spike-in data using either xMAN or Affymetrix probe mapping**. We applied MAT with either xMAN or Affymetrix probe mapping to the spike-in data. xMAN and Affymetrix mapping achieved 100% and 96% True Positive Rate (TPR) at 0% False Discovery Rate (FDR) cutoff, respectively. A MAT prediction is considered correct if the center of the predicted region lies in the actual spike-in fragment. Please note that False Discovery Rate instead of False Positive Rate is used in this ROC-like curve.

We conducted another comparison using a spike-in experiment, in which the position and concentration of every spike-in target was known. The spike-in sample representing a mock ChIP is a mixture of human genomic DNA and 96 clones of approximately ~500 bp, which are 2, 4-, … , 256-fold enriched (12 clones at each concentration) relative to genomic DNA. Genomic DNA without spike-in clones serves as a mock input control. The samples were hybridized to Affymetrix tiling arrays in 5 replicates (GEO accession number GSE5053). With xMAN BPMAP, MAT achieved 100% accuracy for predicting the spike-in clones with 0 false positive and 0 false negative. MAT with Affymetrix BPMAP, however, would yield one false positive prediction (Fig. 3). A scrutiny into this false positive region revealed that most of the probes there had multiple copies in the genome. So by using xMAN BPMAP to control the cross-hybridization effect, MAT successfully eliminated this false positive.

## Conclusions

As many genome sequencing projects continuously update the genome assembly, and high-throughput sequencing/microarray technologies frequently introduce millions of oligonucleotides, algorithm for fast mapping of oligonucleotides to the newest genome is needed. We introduce an intuitive and effective algorithm xMAN, which is optimized for mapping millions of oligonucleotide fragments to the genome simultaneously and is at least an order of magnitude faster than other popular algorithms. It also works on mapping long oligonucleotide probes from NimbleGen and Agilent, and can be further adopted to map the short sequence tags from high throughput sequencing technologies. xMAN can also be used to convert probe genome coordinate between closely related species for analysis, e.g. when Chimpanzee DNA is hybridized to human tiling arrays.

Although the LiftOver program from UCSC can convert genome coordinates, it relies on library files which are not available for all the possible genome assemblies. xMAN, on the other hand, could be used for any coordinate conversion when the query and reference genome sequences are available. In addition, LiftOver only transfers coordinates to a new version without the ability to find other matches of the query DNA sequence. For example, the previously mentioned probe on chromosome 19 which matches ~100 thousand genome locations, will only be converted to a single new genome coordinate by LiftOver.

Most index-based sequence similarity search programs involve two major stages, a heuristic search stage to locate potential similar blocks (anchors) and an alignment stage to combine the anchors. Since xMAN only finds exact matches of short fragments, it simplifies the index-based method by eliminating the second stage. xMAN encodes the query sequences into hash table in RAM and linearly scans through the genome for the exact matches. Query size only affects hash table generation and output writing time, but not genome scanning time. xMAN does require RAM to hash all the query sequences, which is much smaller than the genome size and often available with current computing capacity. Besides, when query sequences are too big, they can be split to multiple files, so xMAN can be carried out sequentially on each smaller query files.

Using xMAN to generate probe mapping BPMAP is important for tiling array data analysis. It not only converts the probe coordinates to newer genome assemblies, but also removes many redundant probes, and allows algorithms such as MAT to consider and control probe cross-hybridization effect. During the BPMAP comparison analysis on ER ChIP-chip data, we not only removed several false-positive ChIP-regions residing in highly repetitive sequences

**Figure 4:**
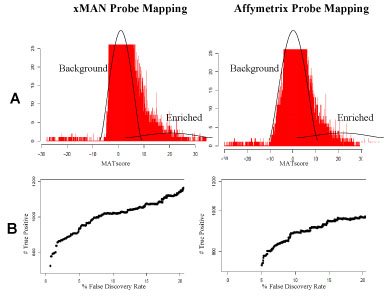
**Estrogen receptor whole-genome ChIP-chip experiment using xMAN or Affymetrix probe mapping**. We applied MAT with xMAN or Affymetrix probe mapping to the estrogen receptor ChIP-chip experiment on Affymetrix human genome 1.0 tiling array set, which consists of 14 arrays covering the non-repetitive human genome at 35 bp resolution. A) MATscore histogram: The standard deviations estimated from the background NULL distribution are 1.07 and 1.09 using xMAN and Affymetrix probe mapping, respectively. Only the bottom part of the histogram was shown. B) Scatter plot of false discovery rate (FDR) versus number of true positives. Under each cutoff, the number of true positive is estimated as the number of positive peaks minus the number of negative peaks; the FDR is estimated as the number of negative peaks divided by number of positive peaks. Under the same FDR cutoff, MAT predicts more true positive peaks using xMAN probe mapping than using Affymetrix probe mapping.

We observed an interesting phenomenon that xMAN BPAMP allows more ChIP-regions to be identified at the same FDR cutoff (Fig. 4). After probe standardization, MAT uses a sliding window approach to calculate a MATscore for each window (e.g. 1KB genomic region) based on the standardized value of all the probes in the window. Assuming the background NULL distribution to be normally and symmetric distributed about the median *m* (often close to 0), MAT estimates the NULL distribution using all the MATscores less than the median. At each MATscore cutoff (*m + x*), the number of peaks below (*m-x*) over the number of peaks above (*m + x*) gives the empirical estimate of FDR. xMAN's more accurate probe mapping and filtering removes the noise from many windows with multiple copy number probes, thereby reducing the overall variance of the MATscore NULL distribution. This leads to a higher signal to noise ratio, and eventually more predictions under the same FDR cutoff.

## Materials and methods

### Hash table for query sequences

xMAN seeks to eliminate the time-consuming disk access operations by creating a query sequence hash table that resides entirely in the computer RAM. Each nucleotide is encoded as two bits, i.e. A: 00; C: 01; G: 10; T: 11 (denoted as BaseIndex), thus any N-mer sequence can be converted into a 2N-bits integer, which is a key in the hash table. In order to avoid encoding ambiguities, all the query sequences are restricted to have the same length. The value of each key in the hash table is a feature associated with the corresponding sequence. The feature can be the X and Y coordinates of a given probe on the microarray, or the position of a sequence in the query file. Redundant query sequences (e.g. if multiple array spots have the same probe sequence) share the same key, with multiple features (e.g. different X and Y coordinates on the array). The memory requirement is proportional to the total size of the query sequence.

### Scanning the genome

xMAN scans the reference genome using a sliding window of size N (query sequence length) at steps of size 1. To accelerate search speed, xMAN also encodes each nucleotide in the genome sequence in 2 bits, and takes advantage of the fastest bit-shifting operation for the encoding, as described below:

Denote the 2N bits integer from the previous window as A

Reset the two left-most bits of A to 0 (i.e. remove the left most base in the previous window)

Left-shift A by 2 bits

Read one more base and add its BaseIndex to A to form the new 2N bits integer for the current window

If this new A is a key in the query hash table, xMAN stores the corresponding chromosome, strand and genomic position.

Step one base 5′ to 3′, and repeat steps 1-5 until the end of the genome

**Figure 5:**
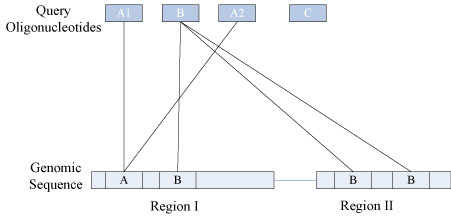
**xMAN mapping example.** In this example, we provide 4 entries in the query (A1, B, A2 and C). A1 and A2 have exactly the same sequence, so they will be mapped to exactly the same genomic position(s). B has 3 copies and C has no match in the genome. The xMAN statistics for this example are: NumUniqSeq: 3; NumSeq.MEntries: 1; NumQueryEntries: 4; NumSeq.MGenomeMatches: 1; NumSeq. NoGenomeMatch: 1; NumTotalEntries: 5.

The scanning is linear in time to the reference genome size. At the end, for each query sequence, xMAN has all its genome copy number and positions, which will be output to a tab-separated values (tsv) file. Query sequences no longer match to the genome are also output as if they are in a chromosome called “NOmatch”. Furthermore, the following xMAN running statistics will be reported. Please see Fig. 5 for an example.

NumUniqSeq: Number of unique sequences in the query

NumSeq.MEntries: Number of sequences with multiple entries in the query

NumQueryEntries: Number of entries in the query file

NumSeq.MGenomeMatches: Number of sequences with multiple genomic matches

NumSeq. NoGenomeMatch: Number of sequences with no match in the genome

NumTotalEntries: Number of total entries in the final xMAN mapping result.

### Software implementation

xMAN is implemented in open source Python, and is freely available at . It requires the query oligonucleotide file and the subject genome file(s). The query file can be in either in Affymetrix binary probe mapping (BPMAP) format or plain-text tab-separated values (tsv) format. The first column in the tsv file must be the sequence and the other columns (optional) could be feature(s) associated with the sequence. The reference genome files must be in Fasta format and can be downloaded from .

## Competing interests

The authors declare that they have no competing interests.

## Authors' contributions

WL carried out the study and drafted the manuscript. JSC and MB performed the molecular biology study. XSL conceived of the study, and helped to draft the manuscript. All authors read and approved the final manuscript.
